# Evaluating the Association of Genetic Polymorphism of Cytochrome p450 (CYP2C9*3) in Gastric Cancer Using Polymerase Chain Reaction-Restriction Fragment Length Polymorphism (PCR-RFLP)

**DOI:** 10.7759/cureus.27220

**Published:** 2022-07-25

**Authors:** Kanishka Uthansingh, Prasanta K Parida, Girish K Pati, Manoj K Sahu, Rabindra N Padhy

**Affiliations:** 1 Gastroenterology, Institute of Medical Sciences and SUM Hospital, Bhubaneswar, IND; 2 Gastroenterology, Srirama Chandra Bhanja Medical College and Hospital, Cuttack, IND; 3 Gastroenterology, Institute of Gastroenterology, Apollo Hospitals, Bhubaneswar, IND; 4 Central Research Laboratory, Institute of Medical Sciences and SUM Hospital, Bhubaneswar, IND

**Keywords:** gastric cancer, genetic polymorphism, helicobacter pylori, cyp2c9*3, risk factors

## Abstract

Background and aim

As a distinguished system, the cytochrome P450 (CYP) enzyme superfamily is involved in the biotransformation of several endogenous and exogenous substances including drugs, toxins, and carcinogens. Reports on the role of CYP enzyme in gastric cancer (GC) from the Eastern region of India are scarce. The present study aimed to evaluate the effect of single nucleotide polymorphisms (SNP) in cytochrome P450 family 2 subfamily C member 9 (CYP2C9*3) among cases with gastric malignancy.

Material and methods

The current study is a cross-sectional observational study carried out among 113 GC cases attending the Institute of Medical Sciences and SUM Hospital, Bhubaneswar, India, and Srirama Chandra Bhanja Medical College and Hospital, Cuttack, India. Two ml of venous blood was collected from the confirmed cases of GC. The samples were subjected to genomic DNA isolation followed by polymerase chain reaction (PCR) and restriction fragment length polymorphism (PCR-RFLP).

Results

The prevalence of both homozygous and heterozygous mutation in GC cases is 4% and 8%, respectively. The overall association of cytochrome P450 family 2 subfamily C member 9 (CYP2C9) mutation in GC cases is 12% whereas 88% were detected as wild/standard type. The mutation CYP2C9 SNP has been seen in *Helicobacter pylori*-infected cases and as well as those without *H. pylori* infection.

Conclusions

The CYP2C9*3 genetic polymorphism might play a significant role as a risk factor for the development of gastric malignancy irrespective of *H. pylori* infection, among the eastern Indian population.

## Introduction

The prevalence of gastrointestinal (GI) tract cancers is gradually becoming a commonplace concern today [1]. Dikshit et al. suggested that stomach cancer (SC) is the leading cause of GI malignancy in Southern India; nevertheless, gastric cancer (GC) is not the measure cause of death. Gastric cancer with legitimate morbidity and residual mortality needs to be well focused on in poor communities [2]. According to a 2018 survey, there are 81,000 cardia-affected GC (CGC) and 8,53,000 cases of non-cardia-affected GC (NCGC) worldwide [3]. Adenocarcinomas mostly affecting the proximal stomach near or including the gastro-oesophageal junction are referred to as cardiac cancer whereas the main portion of the stomach is known as the non-cardia region, hence the non-cardia stomach cancer refers to a tumor in this area. The prevalence of GC differs in different geographical regions because of the varied environmental, genetic, and other risk factors [4-10]. Indeed, the cytochrome (CYP) P450 enzyme plays a crucial role in the metabolism of different compounds such as drugs, food additives, industrial solvents, and pollutants that are ultimately converted into reactive metabolites that could be toxic and carcinogenic to human tissues [11]. Those might even lead to the activation of pro-carcinogens into carcinogens that may increase malignancy risk. Therefore, genetic polymorphism may be co-related with inter-individual differences in cancer susceptibility. The enzyme cytochrome P450 family 2 subfamily C member 9 (CYP2C9) is classified under CYP450 family 2, sub-family C, and polypeptide number 9. As it is known, the gene for CYP2C9 has more than 50 polymorphisms, the specific polymorphism signifying the drug metabolism is called cytochrome P450 family 2 subfamily C polypeptide 9 (CYP2C9*3) [12]. The CYP2C9*3 is an important metabolic enzyme involved in the metabolism of therapeutic drugs and other xenobiotics, such as S-mephenytoin, omeprazole, diazepam, proguanil, propranolol, and certain antidepressants [13]. It has been noticed that approximately 18% of the CYP proteins in liver microsomes are CYP2C9. 

Apart from CYP2C9*2, the CYP2C9*3 also known as 1075A>C (Ile359Leu), is the most widely studied genetic variant, and the single nucleotide polymorphism database (dbSNP) reference identifier for allele location is rs1057910. This specific genetic variant is present in most Caucasians with allelic frequencies of 4% to 10%. Whereas Ustare LA et al. revealed that low rates of CYP2C9 polymorphisms were found; the pattern was similar to that of other Asians, except Indians, and significantly lower than that of Caucasians [14]. A study from India had shown that CYP2C9*3 is present in 4% of Asian-Indian cases [15]. Moreover, the other CYP gene such as CYP2C19 is involved in the detoxification of potential carcinogens [16]. The CYP2C9 gene is present within a cluster of cytochromes P450 genes and is comprised of nine exons and is located with other CYP2C genes in a 500 kb region on chromosome 10q24, and the cluster is comprised of four genes, namely CYP2C8, CYP2C9, CYP2C18, and CYP2C19 [17]. According to the pharmacogenomics of CYP2C9, the enzymatic activity was significantly altered by the mutations in the CYP2C9 gene; individuals carrying non-mutated genes are expected to show high CYP2C9 metabolic capacity. Hence, the relationship between CYP2C9 activity and gastric cancer may be evaluated by analyzing the genotypes involved in the disease. This fact would reveal, whether CYP2C9 enzymatic activity, observed from the activity of specific genotypes, contributes to the development of GC.

The CYP2C9 gene is highly polymorphic with at least 61 variant alleles and multiple sub-alleles [18]. The CYP2C9 gene is not only involved in protein processing and transport but also in the metabolism of compounds such as steroid hormones and fatty acids [19]. Its role in esophageal, antral, and duodenal carcinogenesis and tumor proliferation is well documented. The heterozygous extensive metabolizer (EM) variant is more closely associated with GC compared to the homozygous EM ones [20]. Gastrointestinal tract (GIT) bleeding is more commonly observed in patients on dual therapy with non-steroidal anti-inflammatory drugs (NSAID) and warfarin [21]. The present study aims to evaluate the role of CYP2C9*3 in the development and progression of GC, as there is a lack of information on the role of CYP2C9*3 single nucleotide polymorphism (SNP) in GC.

## Materials and methods

Patients

In the present study non-parametric statistical analyses were performed to examine the percentile of risk involved among patients with GC. The fractional percentile was calculated among the *Helicobacter pylori*-infected and non-*H. pylori*-infected GC patients. A total of 113 consecutive histopathologically proven GC cases were included irrespective of their family history of cancer, gender, and ethnicity. Failure to provide consent, autoimmune disorders, pregnant women, and patients below the age of 18 years were excluded from the study. This study is a triple-centered prospective open-labeled uncontrolled non-interventional cohort study carried out at the Institute of Medical Sciences and SUM Hospital, Bhubaneswar; Srirama Chandra Bhanja Medical College and Hospital, Cuttack; and the Institute of Gastroenterology, Apollo Hospitals, Bhubaneswar. Consecutive cases with histo-pathologically proven GC, attending the outpatient department/inpatient department (OPD/IPD) of these hospitals from November 2019 to November 2020, were enrolled and evaluated prospectively.

*Helicobacter pylori *test

*The H. pylori* infection was assessed by the rapid urease test (RUT) kit (Lenus Medicare and Research (OPC) Pvt. Ltd., Kolkata, India) during the endoscopy procedure whenever a suspected ulcer growth or malignant growth was seen. The sensitivity and specificity of the *H. pylori *RUT test are around 97% and 99%, respectively [22].

Histopathology test

A histopathological test was done to confirm whether the patient is GC-related or non-GC-related. Hematoxylin and eosin staining were done to identify different types of cells and tissues and it provided important information about the pattern, shape, and structure of cells in a tissue sample. Haematoxylin is an organic heterotetracyclic compound 7,11b-dihydroindeno[2,1-c] chromene carrying five hydroxy groups. The GC diagnosis was confirmed by the result observed during the staining.

Sample collections

Two ml of venous blood was collected from all the patients included in the study. All the demographic profiles, laboratory parameters, endoscopy/surgical, and biopsy findings were recorded. The demographic variables such as age, sex, dietary patterns, alcoholic history, smoking history, and infection of *H. pylori* in all confirmed cases of GC were recorded. Moreover, the study participants were prospectively followed up, at every three-month interval for one year.

DNA isolation and polymerase chain reaction-restriction fragment length polymorphism (PCR-RFLP)

The DNA was isolated from heparinized peripheral whole blood from the vials using the salting-out method. In the standard salting-out method, proteins K and RNase are added to them after the lysis of cells. The isolated template DNA samples were stored at 20 °C for further work. The quantity (a minimum of 100ng) and the quality of DNA samples were checked with the Thermo Scientific Multiskan SkyHigh Microplate Spectrophotometer (Thermo Fisher Scientific, Waltham, MA, USA). For the mutational analysis, CYP2C9*3 primers having forward and reverse directions were developed with the help of the Primer3 online software (National Center for Biotechnology Information (NCBI), Bethesda, MD, USA) as shown in Table 1.

**Table 1:** A designed primer with its nucleotide base pairs and digestive enzyme CYP2C9*3: Cytochrome P450 family 2 subfamily C member 9

CYP2C9*3 genetic variant	Sequences	Scale	Purification	No. of bases	Product size	Restriction enzyme
Forward	5’-TGCACGAGGTCCAGAGGTAC-3’	25 nmole DNA Oligo	Standard Desalting	20	131 bp	KpnI
Reverse	5’-AAACATGGATTGCAGTGTAG-3’	25 nmole DNA Oligo	Standard Desalting	20		

The composition for the PCR process for the 15 μl reaction mixture was 100 ng of template DNA, and buffer; the constituents of the buffer were 100 mM Tris having pH 9.0, 500 mM KCl, 15 mM MgCl2 and 0.1% gelatin, 200 μM dNTP, 10 pmol of each primer and 1.0-unit Taq DNA polymerase. The annealing temperature for the PCR program was standardized until reached to get the perfect amplified bands during electrophoresis (Table 2).

**Table 2:** Details of PCR programming CYP2C9*3: Cytochrome P450 family 2 subfamily C member 9, PCR: Polymerase chain reaction

Cycling condition	CYP2C9*3 using kpnI restriction enzyme
Lid temperature	105 ºC
Initial denaturation	95 for 5
Number of cycles	35
Denaturation	95 ºC for 45 seconds
Annealing	58 ºC for 20 seconds
Extension	72 ºC for 20 seconds
Final extension step	72 ºC for 5 minutes
Store forever	4 ºC

**Figure 1:**
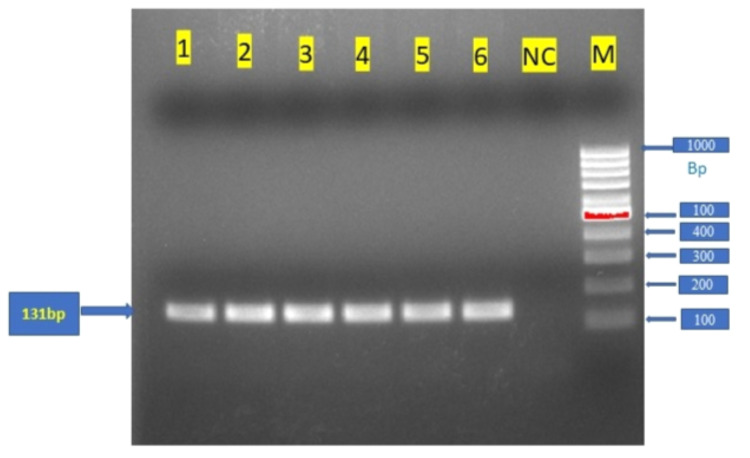
PCR figure showing the confirmatory bands Figure 1 is a screenshot taken from our experimental result. The PCR products of the CYP2C9*3 gene from different GC samples were separated by agarose gel electrophoresis. M: DNA markers (1 kb plus 1000 bp); lanes 1, 2, 3, 4, 5, and 6 are the PCR products of CYP2C9*3 NC: Negative control is a blank column without adding the sample to the reaction mixture CYP2C9*3: Cytochrome P450 family 2 subfamily C member 9, PCR: Polymerase chain reaction, GC: Gastric cancer

The PCR cycle was run with the PCR products containing individual samples in PCR tubes for a specified time set in the PCR machine after standardizing the setting of different annealing temperatures, and the amplified bands were observed in the gel documentation system (Figure 1). The restriction enzymes were developed by the company, and the Thermo Scientific Multiskan SkyHigh Microplate Spectrophotometer was used for the digestion process. The PCR amplified products were digested by 10µ of the restriction enzyme KpnI at 37ºC for three hours, thereafter the genotypes were assessed by 50g/L of agarose gel electrophoresis.

Ethical statement

Written informed consent was obtained from all the concerned patients and the study protocol was approved by the Institutional Ethics Committee of The Institute of Medical Sciences and SUM Hospital, Bhubaneswar (approval no. DMR/IMS-SU/SOA/160129) before inclusion of all the study participants in the study protocol.

## Results

Demographic data analysis

A total of 113 histopathologically proven GC cases were surveyed taking various risk factor for the CYP2C9*3 SNP susceptibility. The gender distribution ratio in the study population, with cases of 52% males and 48% females, was negligible. The study participants were from three states-Odisha with 93% of cases, and the rest were from West Bengal and Jharkhand in eastern India (Table 3*)*.

**Table 3:** Gender and geographic distribution of the participants WB: West Bengal

Gender	Variables	No. of participants (n)	Percentage (%)
	Male	59	52
	Female	54	48
State of origin	Odisha	107	93
	WB	7	6
	Jharkhand	1	6

The demographic data and putative risk factors of the GC cases were statistically analyzed. From the demographic variable analysis, it was ascertained that symptomatically a fraction of 67% of GC patients complained of pain in the abdomen, and 45% of persons had a loss of appetite. Moreover, a fraction of 95% of participants had a rice-based diet, 42% had a wheat-based diet, and a fraction of 63% of participants had a diet history of spicy food.

Out of 113 patients, 24.77% had the habit of regularly chewing tobacco, 30.08% cigarette smoking, and 19.48% were alcoholics. The socio-economic status (SES) as per the Kuppuswamy scale [23] was categorized into upper (I), upper-middle (II), lower-middle (III), upper-lower (IV), and lower class(V). Around 33% of GC cases belonging to upper-middle-class (II) groups were affected by GC followed by the lower (V) group (Table 4).

**Table 4:** Demographic data analysis with different risk variables A percentile is a value in a normal distribution that has a specified percentage of observations. Column 2: Different characteristics of enrolled participants, n: Total number of samples included as cases (column 3), Column 4: Fractional distribution of individual factors for each case in percentage

Variables	Characteristics	Participants (n)	Percentage (%)
Symptoms	Pain Abdomen	76	67.25
	Vomiting	44	38.93
	Diarrhea	8	7.07
	Jaundice	34	30.08
	Loss of appetite	45	39.82
Food habits	Rice-based food	108	95.57
	Wheat-based	48	42.47
	Spicy food	71	62.83
Habits	Alcohol drinking	22	19.46
	Smoking	34	30.08
	Tobacco	28	24.77
Socio-Economic Status (as per Kuppuswamy socio-economic status scale)	Upper(I)	10	9
	Upper Middle (II)	37	33
	Lower Middle (III)	17	15
	Upper lower (IV)	20	18
	Lower(V)	29	25

The distribution of CYP2C9*3 genetic polymorphism with *H. pylori*-positive and negative patients among the GC cases was assessed; a fraction of 36% of* H. pylori*-positive was detected in the total GC cases. Moreover, a fraction of 8% and 4% were CYP2C9*3 heterozygous (AC) and homozygous (AA) mutants, respectively, belonging to CYP2C9*3. These findings revealed that the susceptibility to GC was 12% i.e., 8% for heterozygous and 4% for homozygous mutation. The rest of the 88% were free (negative) from the CYP2C9*3 SNP (Table 5).

**Table 5:** Distribution of H. pylori infection and CYP2C9*3 (1075A>C) genetic polymorphisms CYP2C9*3: Cytochrome P450 family 2 subfamily C member 9, *H. pylori*: *Helicobacter pylori*

Variables	Characteristics	Number of cases (n=113)	Percentage (%)
*Helicobacter Pylori *infection	Positive	41	36.28
	Negative	72	63.71
CYP2C9*3	Wild type (CC)	99	88
	Heterozygous (AC)	9	8
	Homozygous (AA)	5	4

The presence of CYP2C9*3 mutation among *H. pylori*-infected and non-infected cases is summarised in Table 6. Interestingly eight cases had CYP mutation out of 44 *H.pylori-*infected cases whereas six cases were CYP positive among the 72 non-*H.pylori*-infected cases.

**Table 6:** Presence of CYP2C9*3 mutation among H. pylori-infected and non-infected cases *H. pylori*: *Helicobacter pylori, *CYP2C9*3: Cytochrome P450 family 2 subfamily C member 9

H. pylori	No. of Cases	Presence of CYP2C9*3 positivity	Absence of CYP2C9*3 positivity
Present	41	8	33
Absent	72	6	66

A fraction of 12% of subjects of 108 cases consuming a rice-based diet was found to have CYP2C9*3 variants, whereas 10% of subjects of the 48 cases having a wheat-based diet were found to have CYP2C9*3 variants (Table 7*)*. It was observed that a fraction of 20% of subjects of 71 cases who predominantly consume spicy food was found to have the same variant mutation. A fraction of 17.85% of cases with a history of regular tobacco chewing was detected with CYP2C9*3 variants.

**Table 7:** Overall distribution of CYP2C9*3 genetic polymorphism CYP2C9*3: Cytochrome P450 family 2 subfamily C member 9

Diet and other risk factors	Number (n) of cases (total n=113)	Presence of CYP2C9*3 polymorphism	Percentage (%)
Predominantly on a rice-based diet	108/113	13/108	12%
Predominantly on a wheat-based diet	48/113	5/48	10.41%
Consumption of spicy food	71/113	10/71	14.08%
History of regular alcohol drinking	22/113	1/22	4.5%
History of smoking	34/113	4/34	11.76%
History of tobacco chewing	28/113	5/28	17.85%

Follow-up evaluation

The follow-up evaluation was carried out among the GC cases to assess the effect of CYP2C9*3 polymorphism and infection of *H. pylori*. Five cases were lost to follow-up due to miscommunication with the attendees of the patients or by the patients him/herself. After a regular follow-up every three months during the calendar year, it was noticed that a fraction of 11% of cases died, whereas the remaining 89% of cases survived. This suggests that despite improvement in treatment modalities, the prognosis from GC remains to be poor. The one-year survival rate for GC in this study is approximately 89%. 

## Discussion

In the present study, there was an increase in the association between CYP2C9*3 genotype mutation and GC suggesting the possible putative role of causation and progression of GC. Moreover, an association of different genotype polymorphisms with different epidemiological variables in gastric cancer cases was statistically analyzed. Furthermore, the association of different genotype polymorphism, dietary risk factors, history of alcohol abuse, chewing of tobacco, cigarette smoking, infection of *H. pylori*, and dual association of* H. pylori* infection and CYP2C9*3 genetic polymorphism with gastric malignancy was observed. A fraction of 8% had heterozygous genotype (AC) and a fraction of 4% were detected as homozygous mutants (CC) during PCR-RFLP within the CYP2C9*3 variant. The majority of the population had a rice-based diet, out of this, a fraction of 12% of cases had CYP2C9*3 positive results. The above results corroborated with the other study that explored the 30 varieties of genetic polymorphisms in cases with esophageal malignancy in the mutational analysis [24].

**Figure 2:**
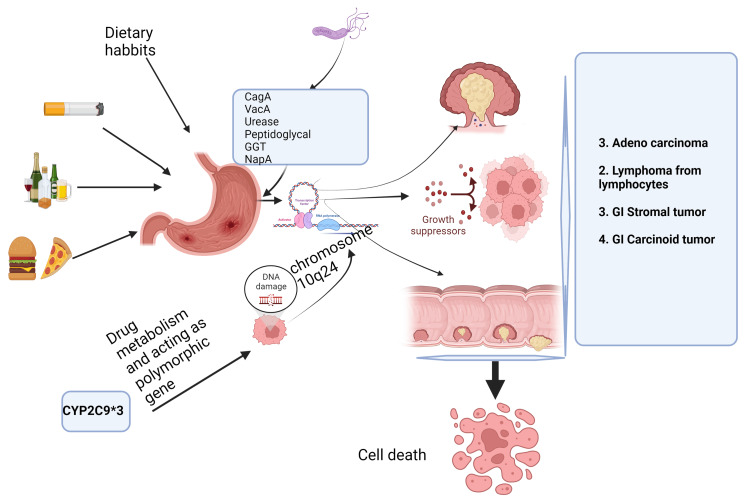
Schematic representation of risk factors associated with GC leading to cell death created is sourced from BioRender CagA: Cytotoxin-associated gene A, VaCa: Vacuolating toxin A, GGT: Gamma-glutamyl transferase, NapA: Neutrophil-activating protein

Different SNPs of CYPs described in the literature so far are CYP1A1, CYP1A2, CYP1B1, CYP2A6, CYP2A13, CYP2B6, CYP2C8, CYP2C9, CYP2C18, CYP2C19, CYP2D6, CYP2E1, CYP2F1, CYP2J2, CYP2S1, CYP3A4, CYP3A5, and CYP4B1 [25]. The association of CYP genetic polymorphisms with cancers of other organs such as the brain, esophagus, stomach, pancreas, pituitary, cervix, melanoma, ovary, kidney, anal canal, and vulva was also explored in a study that is consistent with the present study [26]. Moreover, it was reported that CYP2C9 genetic polymorphisms were not only associated with the risk of developing GC but also with increased risk of the non-aspirin-non-steroidal anti-inflammatory drug (NSAID)-related GI bleeding, as well as interleukin-1b (IL-1b) and tumor necrosis factor-α (TNF- α) polymorphisms [27]. In the current scenario, therapeutics should be targeted for different putative genetic variants such as zeta-chain-associated protein kinase 70 (ZAP70), immunoglobulin lambda-like polypeptide 1 (iIGLL1), cluster of differentiation 79A (CD79A), collagen alpha-3 (VI) (COL6A3), COL3A1, COL1A1, CYP2C18, and CYP2C9, as potential therapeutic regimens for GC [28]. There was no proven association found in any of the CYP2C9 genetic polymorphisms with any type of tobacco-related malignancy [29]. In the present study, it was observed at the 2C9*3 site of cytochrome was susceptible to a fraction of 11% of participants which corroborated with the study by Ghoshal et al. that suggested that the presence of CYP2E1 (96-bp insertion), showed the increased risk of GC even in absence of *H. pylori* infection in the Indian population, whereas CYP1A2 CC or heterozygous mutation (CT) genetic variants were associated with reduced risk for GC [30]. There are different factors leading to GC such as different dietary factors, alcohol and smoking habits, and importantly, genetic mutation and infection of *H. pylori* leading to cell proliferation and cell death (Figure *2*).

A meta-analysis explored that the CYP2C19*2 polymorphism is the putative risk factor for the development of digestive tract cancer [31]. The CYP2C9 genetic polymorphism seems to be related to the development of early esophageal cancer by promoting tumor cell proliferation, which suggests that 2C9*3 is a restricted site for the development of GC. Fundamentally targeted therapy against CYP2C9 genetic variants can be a suitable efficacious therapy in CYP2C9 over-expressed esophageal cancers [32]. Targeted therapy against CYP genetic polymorphism can be an important addition to optimal cancer therapy [33]. The optimal cancer therapy should involve multimodal and multi-targeted therapies including targeted therapy against CYP 450 polymorphisms for better malignancy management and CYP genetic polymorphisms can modulate the risk even in oral cancer [34]. Anticancer drugs with targeted therapy at the p450-mediated tumor metabolism site can be an important addition to anticancer therapy; moreover, targeted therapy against the polymorphic C site can be a promising therapy in future cancer therapy studies [35]. Genetic polymorphism and a deep understanding of patients can be useful in selecting the correct chemotherapeutic drugs for the patients.

The majority of the studies including the present study suggest that esophageal cancer can be associated with mutations of CYP1A1, CYPA1A MspI T/C, CYP1A1 A2455G, CYP1A1T3801C A2455G, and a threefold increased risk for esophageal malignancy observed in patients with CYP2C19PM phenotype variant [36]. Hence, the present study is well elaborated and corroborates different studies that explored the CYP mutation in GC and revealed that 2C9*3 is one of the major restriction sites of CYP, susceptible to GC.

## Conclusions

In conclusion, the present study is the first study of its kind from the Eastern region of India, which vividly studied the association of CYP2C9*3 genetic polymorphisms in GC cases. This is a triple-centric study, hence the results obtained in the study may be referred to study in a multi-centric study. Moreover, there are different SNPs in CYP but, only a single SNP at site 2C9*3 was observed, which may variably affect the results of the present study. Again, the present study included the diseased group but not the healthy group for validation. Although in the present study the investigators have not established a significant association of SNPs of all the associated CYP in GC, we were able to evaluate a single targeted SNP marker named CYP2C9*3 irrespective of infection of *H.pylori. *Further large multicenter studies are required to validate the present results as well as the existing results in the literature so that all the putative genetic biomarkers for GC cases can be explored and can be used as potential genetic biomarkers for the detection of GC in the early stage. Researchers, clinicians, and oncologists from different parts of the world will benefit from the current study as it will give an idea of how cytochrome is one of the key factors causing gastric cancer with the combination of infection of *H. pylori*.
